# Cryptosporidiosis and Giardiasis in Buffaloes (*Bubalus bubalis*)

**DOI:** 10.3389/fvets.2020.557967

**Published:** 2020-10-28

**Authors:** Monally Conceição Costa de Aquino, Sandra Valéria Inácio, Fernando de Souza Rodrigues, Luiz Daniel de Barros, João Luis Garcia, Selwyn Arlington Headley, Jancarlo Ferreira Gomes, Katia Denise Saraiva Bresciani

**Affiliations:** ^1^Estácio de Sá University, Rio de Janeiro, Brazil; ^2^School of Veterinary Medicine Araçatuba, São Paulo State University (UNESP), Araçatuba, Brazil; ^3^Laboratory of Animal Protozoology, Department of Preventive Veterinary Medicine, State University of Londrina, Londrina, Brazil; ^4^Comparative Pathology Shared Resources Laboratory, Masonic Cancer Center, University of Minnesota, Saint Paul, MN, United States; ^5^Laboratory of Animal Pathology, Department of Preventive Veterinary Medicine, State University of Londrina, Londrina, Brazil; ^6^School of Medical Sciences, University of Campinas, São Paulo, Brazil; ^7^Laboratory of Image Data Science, Institute of Computing, University of Campinas, São Paulo, Brazil

**Keywords:** *Cryptosporidium*, *Giardia*, water buffalo, zoonosis, genotypes, assemblages

## Abstract

*Cryptosporidium* spp. and *Giardia duodenalis* infect the gastrointestinal tracts of animals and humans. Both parasite groups are distributed worldwide and cause significant economic losses in animal productivity. Infected hosts presenting with and without clinical manifestations can eliminate infective forms of these protozoa, which are particularly important to One Health. Compared to the published research on cattle, relatively few studies have examined the epidemiology of cryptosporidiosis and giardiasis in buffaloes. This short review describes the global occurrence of *Cryptosporidium* spp. and *G. duodenalis* in buffaloes, including the molecular techniques employed for the identification of species/assemblages and genotypes of these protozoa. Genetic analyses of isolates of *G. duodenalis* and *Cryptosporidium* spp. from various sources (environmental, animal, and human) have been performed to investigate their epidemiology. In buffaloes, the species *Cryptosporidium parvum, Cryptosporidium ryanae, Cryptosporidium bovis*, and *Cryptosporidium suis*-like have been characterized, as well as assemblages A and E of *G. duodenalis*. We demonstrate that buffaloes can be infected by species of *Cryptosporidium* spp. and *G. duodenalis* assemblages with zoonotic potential. Epidemiological studies that utilize molecular biology techniques represent an important resource for efforts to control and prevent the spread of these protozoans.

## Introduction

*Cryptosporidium* spp. and *Giardia duodenalis* (synonym: *Giardia lamblia, Giardia intestinalis*) are unicellular protozoan parasites that infect the intestinal tracts of humans and animals (1–3). Although these parasites possess biological differences, they are frequently discussed together because they share transmission pathways and cause diseases in the gastrointestinal tract (4).

Oocysts and cysts are transmitted *via* the fecal–oral route following direct or indirect contact with the transmissible stages. Possible propagation mechanisms of *Cryptosporidium* spp. and *G. duodenalis* include from animal to animal, from person to person, through zoonotic transmission, *via* contaminated food (5), by water delivery through drinking water, and in the course of recreational activities (6). Insects can also act as mechanical carriers of these protozoa (7). These parasitic forms of *G. duodenalis* and *Cryptosporidium* spp. remain infectious for months in environments with favorable temperature and humidity conditions, which enables the perpetuation of the biological cycle and parasitic spread (4).

Ruminants are frequently considered a major source of excretion of *Cryptosporidium* spp. and *G. duodenalis* for humans (8, 9). Although most studies on this topic have investigated cattle, water buffaloes can also become infected and excrete *Cryptosporidium* spp. oocysts (Table 1) and *G. duodenalis* cysts (Table 2). In general, young buffaloes are more affected by these agents than are older animals (11, 16, 18, 25, 27, 34).

**Table 1:** Occurrence of *Cryptosporidium spp.* in buffaloes worldwide using different diagnostic techniques.

**Country**	**Study period**	**Number of animals**	**Animal age (months old)**	**Positive numbers of buffaloes according to the diagnostic technique**				
				**Diagnostic method**	**Microscopy**	**Immunologic**	**PCR**	**References**
Italy	NA	57	-	ELISA/IFA/PCR	-	8	6^*^	(10)
Italy	2006	347	<1	ELISA	-	51	-	(11)
India	2009–2010	162	<5	ZN/ PCR	62	-	62^*^	(12)
Nepal	2010	81	2–7	ZN/ PCR	30	-	16^*^	(13)
Egypt	2010–2011	538	≤ 4–>4	ZN/ PCR	17	-	17^*^	(14)
India	2009–2010	113	<6	PCR	-	-	41	(15)
Egypt	2011	211	≤ 1–≥1	TRQ/PCR	-	43	75^a^	(16)
India	2009–2012	264	<3	ZN/ PCR	64	-	16^a^	(17)
Australia	NA	476	≤ 24	PCR	-	-	62	(18)
Sri Lanka	2012–2013	297	<6–≥6	PCR	-	-	29	(19)
Thailand	2010–2011	600	≤ 3–≥3	DMSO-MAFS/PCR	34	-	34	(20)
Egypt	2010–2011	466	≤ 3–≥3	ZN/ PCR	6	-	6^*^	(21)
Chine	2012	181	-	PCR	-	-	43	(22)
Brazil	2010	222	5 ≤ 6	PCR	-	-	107	(23)
India	2012–2013	246	≤ 3–≥3	ZN/ PCR/ S	91	-	6^a^	(24)
Egypt	2014	130	<2–>12	ZN/ PCR	16	-	4^a^	(25)
Australia	2015	100	24 ≤ 60	qPCR	-	-	21	(26)
India	2014–2016	83	≤ 3–≥3	/PCR	-	9	1^*^	(27)
Brazil	NA	122	<12	Nested PCR/RFLP	-	-	16	(28)
Australia	2017	313	-	PCR	-	-	42	(29)

* *PCR was performed only on positive samples by a previous screening method*.

a *PCR was performed on only some of the positive samples by the screening method*.

*PCR, Polimerase Chain Reaction; ZN, Ziehl-Neelsen; ELISA, Enzyme-Linked Immunosorbent Assay; IFA, Immunofluorescence Assays; TRQ, Test RIDA®QUICK; DMSO- MAFS, DMSO- DMSO- modified acid-fast stain; IM, Imunofluorescent Microscopy; S, Sheather*.

*NA, not available*.

**Table 2:** Occurrence of *G. duodenalis* in buffaloes worldwide using different diagnostic techniques.

**Country**	**Study period**	**No of. animals**	**Animal age (months)**	**Positive numbers of buffaloes according to the diagnostic technique**				
				**Diagnostic method**	**Microscopy**	**Immunologic**	**PCR**	**References**
Italy	NA	57	-	ELISA/IFA/PCR	-	15	8^*^	(10)
Italy	2006	347	<1	ELISA	-	63	-	(11)
Australia	NA	476	≤ 24	PCR	-	-	62	(18)
Sri Lanka	2012–2013	297	<6–≥6	PCR	-	-	2	(19)
Egypt	2011	211	≤ 1–≥1	Test RIDA®QUICK/PCR	10	-	10	(30)
India	2012	22	-	IMS/DFA	-	9	-	(31)
Egypy	2013–2014	100	1 <4	DSM/ FEST / S/PCR	25	-	25	(32)
India	2014–2016	83	≤ 3–≥3	IM/PCR	-	9	1	(27)
Brazil	2016	183	≤ 6	PCR	-	-	12	(33)
Australia	2017	313	-	PCR	-	-	14	(29)

* *PCR was performed only on positive samples by a previous screening method*.

*PCR, Polymerase Chain Reaction; IMS, Immunomagnetic separation; DFA, Direct immunofluorescence; ELISA, Enzyme-Linked Immunosorbent Assay; IFA, Immunofluorescence Assays; Direct smear method; Formalin-ether sedimentation technique; IM, Immunofluorescent microscopy; S, Sheather; RIDA®QUICK. NA, not available*.

*Cryptosporidium* spp. and *G. duodenalis* cause mild and/or moderate disease, with diarrhea being the main clinical sign. There are few reports describing the economic burden of *Cryptosporidium* spp. and *G. duodenalis* infection in ruminants. The costs of treatment, reduced feed conversion, production inefficiency, and the involvement of many animals in the herd cause considerable economic losses on farms worldwide (25, 35). Dairy calves determined to be negative for *Cryptosporidium* or *G. duodenalis* by immunofluorescence microscopy showed higher average daily gain than did calves that were positive for these parasites (36).

The application of molecular approaches for the identification of these two parasites has led to significant advances in knowledge regarding the epidemiology of these protozoans, with different species being characterized (37).

Molecular analysis of human and animal isolates has demonstrated that *G. duodenalis* is a complex species, with eight assemblages being recognized. Assemblages A and B are observed in humans and other mammals, assemblages C and D are specific to dogs and other canids, assemblage E is found in hoofed animals, including livestock, assemblage F is detected in cats, assemblage G is found in rodents, and assemblage H is observed in pinnipeds (38).

To date, at least 38 species of *Cryptosporidium* spp. have been recognized by molecular characterization (39–41), with 73 genotypes (42–44) and 17 species having been identified in humans (45).

*Cryptosporidium* spp. and *G. duodenalis* may represent a problem to the buffalo industry due to their economic cost and the risk of human exposure associated with oocysts and cysts eliminated in the environment by infected hosts. Thus, in this review, we demonstrate the global occurrence of these protozoa, emphasizing the importance of the molecular characterization of their species/assemblages and genotypes reported in buffaloes.

## Diagnostic Methods

Cryptosporidiosis and giardiasis can be diagnosed by a wide variety of parasitological, serological, and molecular techniques (4, 46–48).

Regarding the parasitological diagnosis technique, the identification of the morphological structures of the *Cryptosporidium* spp. oocysts and *G. duodenalis* trophozoites or cysts is important, with *G. duodenalis* being diagnosed by direct microscopic observation of the trophozoites or cysts in feces. Trophozoites can be observed by direct microscopic examination of freshly collected samples, which are immediately prepared with saline solution at 37°C (49). Concentration techniques, such as zinc sulfate (50), sucrose (51), formalin (52), and the “three fecal test” (TF test) (53), are recommended before the observation of cysts since these methods promote an increase in diagnostic sensitivity. Centrifugal flotation with zinc sulfate is one of the most commonly employed methods for the detection of *G. duodenalis* from fecal samples; however, sucrose flotation works adequately well, and it is generally employed in ruminant samples since oocysts of *Eimeria* spp. and *Cryptosporidium* spp. may also be encountered (4).

Various staining procedures can be used to differentiate between *Cryptosporidium* spp. oocysts and *G. duodenalis* cysts from coexisting protists and for excluding similarities from environmental or fecal debris. Smear preparations stained by the trichome and iodine or iron hematoxylin methods can be utilized to assist in the detection of various stages of *G. duodenalis* (49). The most frequently used routine techniques for examining stained slides to identify *Cryptosporidium* spp. are the modified Ziehl–Neelsen (54, 55), modified Kinyoun (56, 57), and methylene blue safranin (58, 59) stains. Additionally, the negative coloring observed with the malachite green technique (53, 60) can be used. The centrifugal flotation technique with Sheather's solution (61), which uses brightfield or phase contrast optical microscopy, can be employed to visualize oocysts (8). However, differentiating between *Cryptosporidium* species/genotypes using microscopy is not possible because oocysts are similar in shape and overlap in size (37).

Immunological methods have higher sensitivity and specificity than light microscopy in characterizing diverse types of samples (49, 62, 63). Monoclonal antibodies targeting antigens in fecal samples are sensitive diagnostic methods (64, 65). Antigens of *Cryptosporidium* spp. and *G. duodenalis* in fecal contents can be detected by direct immunofluorescence assay (63, 66), ELISA (67–69), and rapid solid-phase qualitative immunochromatography assays (67). Some immunoenzymatic tests can be employed to detect infection in animals that are not eliminating cysts in the feces (70), which means that these tests can be utilized to screen large numbers of animals rapidly (67). However, these methods have the disadvantage of not being able to identify species or genotypes (45).

The application of molecular techniques has resulted in expanded knowledge regarding the taxonomy and epidemiology of *Cryptosporidium* spp. and *G. duodenalis*. Molecular diagnostics are widely used to differentiate *Cryptosporidium* spp. and *G. duodenalis* species or genotypes (35). Therefore, such methods as polymerase chain reaction (PCR), real-time PCR, or multiplex PCR together with DNA sequencing can identify species/assemblages with high sensitivity and specificity, and these techniques can be employed to identify sources of transmission as well as the zoonotic potential of these two parasites (71–73).

Genotyping targeting the small subunit of the ribosomal RNA gene (*ssu* rRNA) aligned with PCR and sequencing the restriction fragment polymorphism (RFLP) are sensitive molecular tools used in the detection of *Cryptosporidium* spp. DNA in fecal and environmental samples (74, 75). Other genetic regions have also been studied, such as the 70-kDa heat shock protein (*hsp*70), thrombospondin-related adhesive proteins, *Cryptosporidium* spp. oocyst wall protein (*cowp*), and actin genes (76).

The 60-kDa glycoprotein (*gp60*) is located on the surface of the apical region of invasive stages of the parasite. This gene is a highly polymorphic marker that is widely used in *Cryptosporidium* spp. subtyping because of its high polymorphism and relevance to parasite biology, and the use of *gp60* subtyping has identified human-specific, animal-specific, and zoonotic subtypes for *Cryptosporidium parvum* species-specific subtypes (37). More than 20 *gp60* genotype families of *C. parvum* have been described, including several subtypes within each family, with families IIa and IId being identified from ruminants and humans and recognized as zoonotic. The IIa family and subtype IIaA15G2R1 are frequently identified in calves (3, 77).

Many genetic markers have been employed for the molecular characterization of *G. duodenalis* using degenerated primers, such as the *ssu* rRNA, glutamate dehydrogenase (*gdh*), elongation factor 1-alfa (*el1-*α), triose-phosphate isomerase (*tpi*), or genes uniquely associated with the parasite, such as beta-giardin (*bg*) (78). The *ssu* rRNA gene is strongly conserved and can be utilized to identify *G. duodenalis* assemblages, but it is of little use for studies where genetic variation within assemblages needs to be determined (78). Consequently, the *tpi, bg*, and *gdh* sequences are employed because polymorphism enables the characterization of the genetic heterogeneity of this parasite (79) as well as its zoonotic potential (78, 80). However, molecular methods are mostly restricted to research and specialized laboratories (37).

## Epidemiology

Infections by *Cryptosporidium* spp. and *G. duodenalis* in water buffalo (*Bubalus bubalis*) have been less thoroughly well-investigated compared with other bovid species. Tables 1, 2 summarize the occurrence of these protozoa in different buffalo populations.

According to the findings described in Table 1, the infection rates of cryptosporidiosis reported in buffaloes ranged from 1.3% (Egypt) to 48.2% (Brazil). However, the epidemiology of giardiasis has been more heavily studied in cattle, with few reports describing *G. duodenalis* infections in buffaloes (Table 2), and a prevalence ranging from 1.3% (Sri Lanka) to 10.5% (India) was reported.

Differences in the prevalence of infection for *Cryptosporidium* spp. and *G. duodenalis* in buffaloes are common among epidemiological studies. The discrepancies in the reported infection rates may be attributed to the differences in environmental conditions, management practices, time between collection and processing of fecal samples, the diagnostic techniques used, age, and the number of animals analyzed in each study.

High population density, with more than 100 buffaloes being raised on a farm, intensive management systems where buffalo calves are reared at high density (11), poor or inadequate hygiene, concrete floor (81), canalized or underground water sources (16, 81), and the winter period (24, 81), as well as rainy periods (17), were relevant risk factors associated with the occurrence of infection by *Cryptosporidium* spp.

In preweaned calves, *C. parvum* causes high morbidity associated with profuse diarrhea, lethargy, anorexia, and dehydration (82). The clinical signs are evident 3–5 days after infection, and the duration of the clinical signs can vary between 4 and 18 days (83). Diarrhea is accompanied by the excretion of large amounts of oocysts, which can be subsequently dispersed within the environment (82).

Some studies have observed an association between infection by *Cryptosporidium* spp. and diarrhea in buffalo calves (17, 24, 34, 81), with a higher occurrence of infection being observed in the 1st months of age (24, 25, 34).

In calves of cattle and buffaloes from the Mumbai region of India, diarrheic feces showed a higher prevalence of *Cryptosporidium* spp. than did apparently normal feces. The highest prevalence was observed in the youngest group, declining gradually with advancing age, with the lowest prevalence being observed in adults, indicating an inverse correlation between the prevalence rate and age of the host (24). The presence of *Cryptosporidium* spp. oocysts in the feces of water buffaloes without clinical signs was verified at 6 weeks of age, which suggests that asymptomatic individuals are potential sources of infection (84).

Mixed infections by *Cryptosporidium* spp. and *G. duodenalis* in buffalo were detected by real-time PCR in 36% of animals (cattle and buffaloes) from Egypt (30). These parasites may be detected together in calves with diarrhea, and coinfection with other pathogens has also been reported (84).

In Egypt, the occurrence of *Cryptosporidium* spp. was 9.5% (17/179) in calves of buffaloes and absent in 359 adult water buffaloes (14). In some studies, *C. parvum* was identified only in buffaloes <6 months of age (18, 34). However, in Northeast Australia, all samples positive for *C. parvum* were isolated from adult buffaloes (2–5 years), indicating that this species can also commonly infect adult buffaloes (26). In Thailand, *C. parvum* infection was identified in all age groups, with no significant difference in the infection rates being observed among the age groups assessed (20).

The epidemiology of giardiasis has been more studied in cattle, and there are few reports of *G. duodenalis* infections in buffaloes (Table 2). In Pakistan, 2.7 × 10^2^ cysts per gram of feces of *G. duodenalis* were excreted in fecal samples with normal consistency. Additionally, in cases of diarrhea, the intensity of elimination was higher (4.3 × 10^3^ cysts per gram of feces). In that same study, buffalo calves (≤ 1 year) had significantly higher cyst prevalence (85).

The prevalence of *G. duodenalis* in Australia was 13% (62/476) with the identification of assemblages A (11.8%) and E (1.2%), respectively.

In an overpopulated province of Egypt, *G. duodenalis* cyst/trophozoites were observed in 20% of fecal samples from people and in 25% of buffalo calves by nested PCR. Contact with calf manure and inappropriate personal hygiene practices, such as hand washing and changing shoes after handling the animals, were considered to be risk factors significantly associated with giardiasis (32).

## Molecular Aspects

Molecular diagnostic tools play an important role in understanding the transmission of *Cryptosporidium* spp. and *G. duodenalis*, mainly due to the existence of many morphologically identical species and genotypes within both groups of protozoa. Thus, these tools are required for the differentiation of these species and genotypes (86).

Although the molecular epidemiology of *Cryptosporidium* spp. and *G. duodenalis* is well-studied in cattle, studies are scarce in buffaloes. Cattle are commonly infected by four species of *Cryptosporidium*, with *C. parvum* being predominant in preweaned calves, *Cryptosporidium bovis* and *Cryptosporidium ryanae* predominant in the postweaning phase, and *Cryptosporidium andersoni* predominant in adults (45, 75, 87). However, differences in the occurrence of different species according to age groups in buffaloes were not observed.

**Figure 1:**
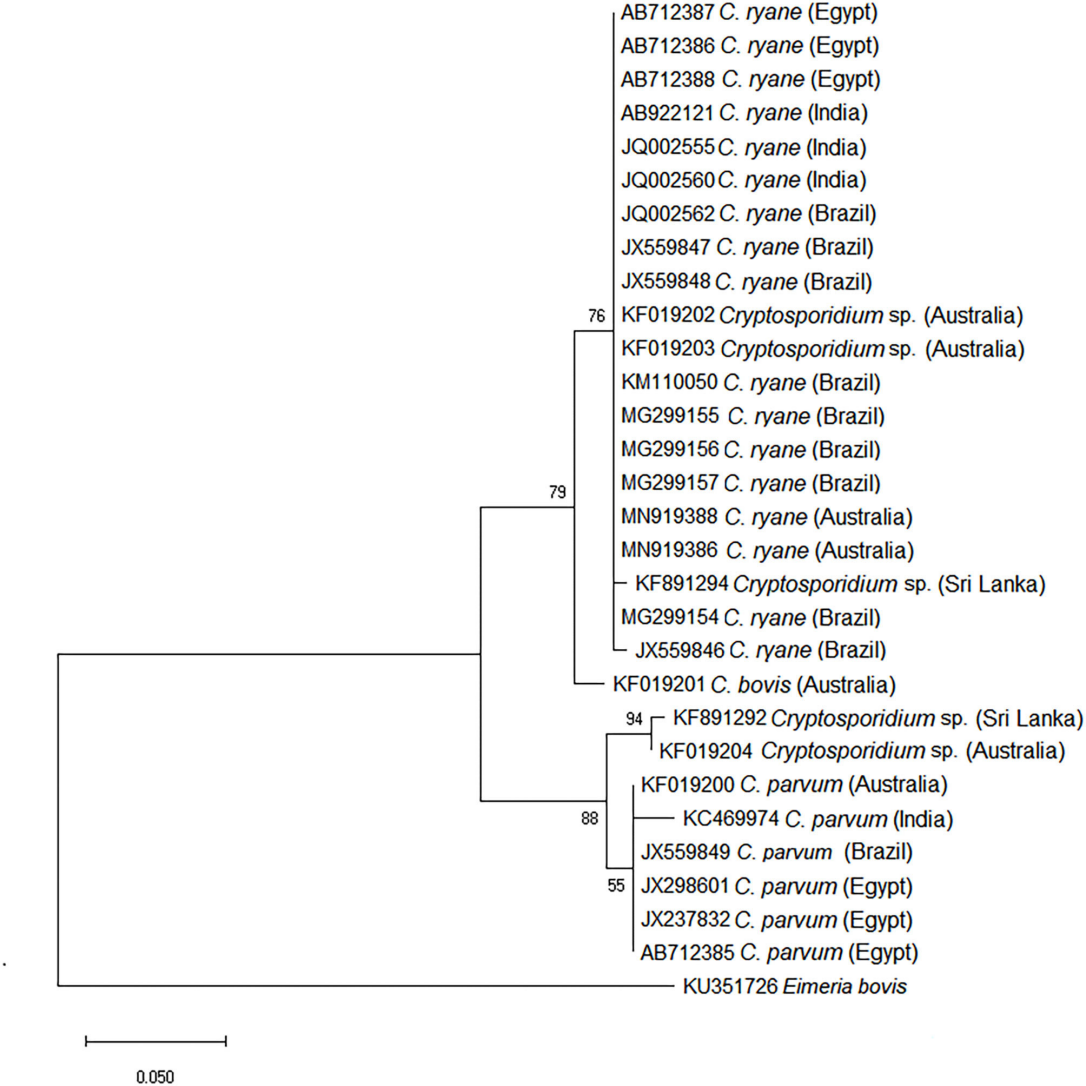
Phylogenetic tree of species of *Cryptosporidium* using the *ssu* rRNA gene obtained from buffalo samples. The evolutionary history was inferred by using the maximum likelihood method and the Tamura–Nei model using MEGA software.

Previous studies have determined the species of *Cryptosporidium* infecting buffaloes in different countries; *C. parvum, C. ryanae, C. bovis*, and a genotype similar to that of *Cryptosporidium suis* were identified (Table 3). Phylogenetic analysis using a range of sequences of the *ssu* rRNA gene of *Cryptosporidium* spp. retrieved from the GenBank database from various geographical regions supports the classification of the species and genotypes of *Cryptosporidium* spp. in buffaloes (Figure 1).

**Table 3:** Species and subtypes of *Cryptosporidium* in buffaloes worldwide.

**Country**	**Number of animals**	**Genes**	**Species of *Cryptosporidium***	***gp60* genotype**	**References**
Italy	57	*ssu rRNA*	*C. parvum*	-	(10)
India	162	*ssu rRNA*	*Cyptosporidium* spp.	-	(12)
Nepal	81	*ssu rRNA*	*C. ryanae*	-	(13)
India	113	*ssu rRNA*	*Cyptosporidium* spp.	-	(15)
India	264	*ssu rRNA*	*C. parvum*	-	(17)
Australia	476	*ssu rRNA*	*C. bovis. C. parvum. C. suis like. C. ryanae*	-	(18)
Egypt	211	*ssu rRNA, gp60*	*C. bovis.C. parvum. C. ryanae*	IIaA15G1R1(1). IIdA20G1 (1)	(16)
Egypt	538	*ssu rRNA, gp60*	*C. parvum. C. ryanae* (10)	IIaA15G1R1 (5). IIdA20G1 (2)	(14)
Egypt	466	*ssu rRNA, gp60*	*C. parvum. C. ryanae*		(21)
Sri Lanka	297	*ssu rRNA, gp60*	*C. ryanae. C. suis like*	-	(19)
Thailand	600	*ssu rRNA*	*C. parvum.C. ryanae*	-	(20)
Chine	181	*ssu rRNA*	*C. bovis. C. ryanae*	-	(22)
Brazil	222	*ssu rRNA, gp60, cowp*	*C. parvum. C. ryanae. C. suis like*	IIaA15G2R1(2)	(23)
Egypt	130	*ssu rRNA, gp60, cowp*	*C. parvum*	IIdA20G1 (4)	(25)
India	246	*ssu rRNA*	*C. parvum. C. ryanae*	-	(24)
Australia	100	*ssu rRNA, gp60*	*C. bovis. C. parvum*	IIaA18G3R1(5) IIdA19G1 (4). IIdA15G1 (1)	(26)
India	83	*ssu rRNA, gp60*	*C. bovis. C. parvum*	IIdA15GR1 (1)	(27)
Brazil	122	*ssu rRNA, gp60*	*C. parvum. C. ryanae*	IIdA20G1R1 (4)	(28)
Australia	313	*ssu rRNA*	*C. ryanae*	-	(29)

*ssu rRNA, Small Subunit of the ribosomal RNA gene; gp60, 60 kDa glycoprotein gene; cowp, Cryptosporidium spp. oocyst wall protein*.

*Cryptosporidium parvum* is the most frequently reported zoonotic species that infects humans and ruminants worldwide (8). Numerous studies have been conducted to subtype *C. parvum* in farm animals, especially calves, to characterize the transmission dynamics and zoonotic potential of *C. parvum*. Table 3 shows that most of the studies using *gp60* and sequencing analysis have observed that buffaloes are commonly infected with the IIa family. Additionally, family IId has been found in buffaloes in Egypt (14, 21, 25, 30) and India (27).

*Cryptosporidium ryanae* infects ruminants and has been widely reported in calves from different age groups worldwide (13, 14, 18, 19, 22–24, 28, 29). Data from recent studies suggest that there is a host-adapted *C. ryanae* in water buffaloes, as reflected by the high occurrence of this species in these animals (13, 23, 28). Some findings show that the genotypes of *C. ryanae* found in water buffaloes are different from those identified in cattle (13, 18, 22, 23). Therefore, further research is warranted to determine the taxonomic status and host specificity of these genotypes found in water buffaloes (19).

*Cryptosporidium bovis* is predominantly a parasite of livestock and has been reported in buffaloes on several occasions (16, 18, 22, 26, 27). Due to the lower occurrence of this species compared to other species of *Cryptosporidium* spp. reported in buffaloes, the possibility of a higher affinity of *C. bovis* for *Bos taurus* or *Bos indicus* was considered than for *B. bubalis* (18).

The *ssu* rRNA sequences of *C. suis*-like obtained from buffaloes in Brazil (23), Australia (18), and Sri Lanka (19) were similar to those previously identified in cattle (88–90). Based on the analysis of other genetic markers, such as *hsp* 70 and *actin*, it was observed that the sequences generated were clearly different from those of *C. suis* (23).

Regarding *G. duodenalis* infection in buffaloes, the first molecular characterization of *G. duodenalis* was performed in Italy with the detection of assemblage E and assemblage A, subassemblage AI. Based on this finding, buffaloes were suggested to contribute to environmental contamination due to the elimination of potentially infectious parasitic cysts in humans (10).

There are a limited number of studies on giardiasis in buffaloes, and in most of these, the dominant assemblage identified was assemblage E (Table 4); however, zoonotic assemblage A was reported to be predominant in buffaloes from Australia (18). The predominance of this assemblage was unexpected (18).

**Table 4:** Assemblages and sub-assemblages of *G. duodenalis* in buffaloes worldwide.

**Country**	**Study period**	**Number of animals**	**Animal age (months)**	**Genes**	**Assemblages**	**Sub-assemblages**	**References**
Italy	NA	57	-	*bg*	A. E	A1	(10)
Australia	NA	476	A/c	*tpi*	A. E	-	(18)
Sri Lanka	2012–2013	297	<6–≥6	*tpi*	E	-	(19)
Egypt	2011	211	A/c	*bg*. *gdh*. *tpi*	E	-	(30)
India	2012	22	-	*gdh*	-	-	(31)
Egypt	2013–2014	100	1–4	*ssu* rRNA	-	-	(32)
India	2014–2016	83	A/c	*ssu* rRNA. *tpi*	A	-	(27)
Brazil	2016	183	≥6	*ssu* rRNA. *bg. gdh. tpi*	E	-	(33)
Australia	2017	313	-	*bg*. *gdh*	E	-	(29)

*ssurRNA, Small Subunit of the ribosomal RNA gene; bg, β-giardin gene; gdh, Glutamate dehydrogenase gene; tpi, triosephosphate isomerase gene; A/c, Adults/calves; NA, not available*.

Few studies have typed assemblage A isolates in buffaloes, but subassemblage AI was the most common (10). Studies on the prevalence of *G. duodenalis* in cattle observed the existence of different subtypes of assemblage E identified by the multilocus genotyping scheme (MLG) based on the *bg, gdh*, and *tpi* genes (91, 92). The differences in the distribution of *G. duodenalis* assemblage E MLGs from cattle likely indicate geographical segregation (91).

## Public Health Impact

Due to the close relationship of these protozoans with poor basic sanitation and low population purchasing power, *Cryptosporidium* spp. and *G. duodenalis* were included in the World Health Organization's Neglected Diseases Initiative (93).

Water transmission of *Cryptosporidium* spp. and *G. duodenalis* is particularly important (94), with outbreaks being reported in several countries (95, 96). Most people in undeveloped countries do not have access to good quality water. Consequently, the contamination of drinking and bathing water with these two pathogens and the use of sewage for agricultural purposes pose a serious threat to millions of individuals worldwide (97).

The transmission of *Cryptosporidium* spp. and *G. duodenalis* has also been associated with the consumption of contaminated food due to the use of fertilizers based on animal feces (manure), the use of untreated water for the irrigation of farmland, and runoff from feedlots. In addition, food can be contaminated during harvesting, packaging, transportation, and preparation under unhygienic conditions (98).

Human infections by *Cryptosporidium* spp. are commonly caused by *Cryptosporidum hominis* and *C. parvum* (1, 99), although other species have previously demonstrated zoonotic potential, such as *Cryptosporidum ubiquitum* (100), *Ctenocephalides canis* and *Ctenocephalides felis* (101–103), *Crytosporidium muris* (104, 105), *C. suis* (106), *Crytosporidium cuniculus* (107, 108), and *Crytosporidium meleagridis* (109–111).

Assemblages A and B of *G. duodenalis* can be observed in both humans and animals. Less frequently, other *G. duodenalis* genotypes have been only occasionally detected in humans (112, 113), with the possible exception of assemblage E, which has recently been detected in substantial numbers of human cases in rural areas of Egypt (25, 30, 114), Brazil (115), and Australia (116).

Cattle are recognized as a major contributor to zoonotic sources because the species and genotypes of *Cryptosporidium* spp. and *G. duodenalis* that infect humans have also been isolated from cattle (35).

Especially in developing countries, these parasites cause diarrhea in malnourished children under 5 years of age (117). Cryptosporidiosis in immunocompetent individuals is considered self-limiting, but with the appearance of the acquired immunodeficiency syndrome (HIV), opportunistic infections have been associated with more serious and even fatal clinical manifestations in immunosuppressed individuals (35, 118, 119).

The common occurrence of *C. parvum* subtypes IIaA15G1R1 and IIdA20G1 in buffaloes and humans in Egypt, respectively (16, 25), highlights the importance of zoonotic transmission with a special emphasis to the potential role of these animals as significant reservoirs of infection to humans.

In humans, most of the infections related to *G. duodenalis* are asymptomatic; however, acute or chronic diarrhea, dehydration, abdominal pain, nausea, vomiting, and weight loss can occur (120, 121). Most cases occur in individuals who are under 5 years of age, malnourished, and immunocompromised (80). Functional intestinal disorders, such as irritable bowel syndrome, can be associated with a previous infection (122). Infected children may show developmental delays, decreased cognitive function and nutritional status (123).

Among the assemblages of *G. duodenalis*, assemblages A and B have the broadest host specificity, having been known to infect humans and various other mammals (124). Both assemblages can be transmitted zoonotically, indicating a significant public health impact, and there are reports of farmers being infected with these assemblages (37). However, assemblage B has not been reported in buffaloes (124).

Assemblage E is considered species-specific; however, there are reports of humans being infected with this assemblage, and it was suggested that this assemblage might present an emerging anthropozoonotic cycle (30, 114–116, 125).

In Ismailia, one of the most densely populated provinces of Egypt (regarding livestock and people), fecal samples from children, cattle, and buffaloes were examined for the detection of *G. duodenalis* coproantigens and analyzed by MLG (*bg, gdh*, and *tpi*). Assemblage B was dominant in humans, while assemblage E was more frequent in ruminants and was detected in two children, indicating a potential route for anthropogenic infection. It was also observed that drinking tap water, but not contact with animals, was associated with an increased risk for children to be infected (30).

In Egypt, the contact of people with buffaloes and their feces was identified as a risk factor for the prevalence of infection by *G. duodenalis*, drawing attention to zoonotic transmission (32).

In rural regions of India, *G. duodenalis* and *Cryptosporidium* spp. have been detected in humans, buffaloes, cattle, goats, sheep, dogs, and water sources (tube wells and lakes). These findings show preliminary evidence of the diversity of possible transmission routes and help to elucidate the distribution of these parasites in coexistence with humans and animals and their water sources (31).

## Control and Prevention

To date, there is still no vaccine or drug that is effective in the treatment of ruminants with cryptosporidiosis, which makes the control of infection difficult. Therefore, the best strategy is to adopt management measures to reduce the spread of the disease in herds (126, 127).

The recommended methods for controlling *Cryptosporidium* spp. infection in ruminants are similar to those recommended for other protozoa and coccidia. Consequently, good breeding practices should be employed out, such as separating animals with diarrhea, cleaning the premises before the animals are introduced, removing and eliminating fecal content or wet garbage, cleaning feeders and drinking fountains, developing strategies to reduce humidity in facilities, and providing adequate supplies of colostrum to neonates (128, 129).

The survival time of oocysts can be reduced by freezing, desiccation, exposure to ultraviolet light, and variations in hydrogen potential (pH) and temperature (74). *Cryptosporidium* spp. oocysts are sensitive to ozone, at temperatures of 55°C for 30 s or 70°C for 5 s (130). Thus, viable solutions for disinfecting the environment consist of 10% formaldehyde for 18 h and 5 or 50% ammonia for 30 min (131–133).

In some studies, there has been a reduction in the excretion of *Cryptosporidium* spp. oocysts in cattle calves treated with azithromycin (134, 135). Other studies with intestinal microbiota found that fiber deprivation in the diet increases the susceptibility of mice to cryptosporidiosis, and there is a need for research to confirm this possibility in other animal models (136).

Such compounds such as paromomycin (9), albendazole (137, 138), and fenbendazole (9, 139) can be used for the treatment of giardiasis in cattle calves. The action of probiotics against *G. duodenalis* was primarily investigated *in vitro*. Probiotics that modulate the immune response have a beneficial effect on the composition of the intestinal microbiota and minimize parasite–host interactions (140, 141).

There is no vaccine available for the prevention and control of giardiasis in ruminants (142). Prophylactic measures are similar for the two protozoa and should include complete cleaning and disinfection of housing facilities using such products as ammonia, chlorine dioxide, hydrogen dioxide, and ozone. Additionally, maintaining a dry environment inside buildings may hinder the development of parasites (46, 143).

Minimizing the spread of *G. duodenalis* infections among ruminants is a considerable challenge (137). Thus, environmental disinfection associated with drug therapy is recommended (9, 137, 138). The daily removal of feces in the stable, pens, and surroundings is also important. Additionally, it is essential to ensure adequate intake of colostrum by newborns for the establishment of passive immunity (37). These measures need to be incorporated into the management of the herd with the main priority of adopting good health practices.

## Conclusion

Based on recently published research, we demonstrate the global occurrence of *Cryptosporidium* and *G. duodenalis* in buffaloes from different geographic regions. Although these parasites possess biological differences, they are frequently discussed together because they share transmission pathways and cause diseases in the gastrointestinal tract. Water buffaloes can also become infected and excrete *Cryptosporidium* spp. oocysts and *G. duodenalis* cysts. In general, young buffaloes are more affected by these agents than are older animals. Infected hosts presenting with and without clinical manifestations can eliminate the infective forms of these protozoa in the environment. Cryptosporidiosis and giardiasis can be diagnosed by a wide variety of parasitological, serological, and molecular techniques. The application of molecular approaches for the identification of these two parasites has led to significant advances in knowledge regarding the epidemiology of these protozoans, with different species being characterized. The common occurrence of these parasites in both buffaloes and humans highlights the potential role of zoonotic transmission in the epidemiology of cryptosporidiosis and giardiasis. However, molecular methods are mostly restricted to research and specialized laboratories, and further research is warranted to determine the taxonomic status and host specificity of these genotypes found in water buffaloes. We recommend that measures need to be incorporated into the management of the herd with the main priority of adopting good health practices and highlighting the importance of using molecular tools to identify species/genotypes for a better understanding of the epidemiology of these protozoa relevant in public health.

## Author Contributions

All authors have made substantial contributions to the conception of this work and critically reviewed for important intellectual content. We have also collectively approved the final version to be published and unanimously agreed with all aspects of this work, ensuring that issues related to the accuracy and integrity of any part of this work are properly investigated and resolved.

## Conflict of Interest

The authors declare that the research was conducted in the absence of any commercial or financial relationships that could be construed as a potential conflict of interest.
